# Comparing glycaemic benefits of Active Versus passive lifestyle Intervention in kidney Allograft Recipients (CAVIAR): study protocol for a randomised controlled trial

**DOI:** 10.1186/s13063-016-1543-6

**Published:** 2016-08-22

**Authors:** Joanne Wilcox, Chantelle Waite, Lyndsey Tomlinson, Joanne Driscoll, Asra Karim, Edward Day, Adnan Sharif

**Affiliations:** 1 Department of Nephrology and Transplantation, Queen Elizabeth Hospital, Birmingham, UK; 2 Department of Nutrition and Dietetics, Queen Elizabeth Hospital, Birmingham, UK; 3 National Addiction Centre, Institute of Psychiatry, Psychology and Neuroscience, King’s College London, London, UK; 4 School of Immunology and Inflammation, University of Birmingham, Birmingham, UK

**Keywords:** Kidney transplantation, Post-transplantation diabetes, New-onset diabetes after transplantation, Glycaemic metabolism, Weight gain, Cardiometabolic, Lifestyle intervention, Lifestyle modification, Behavioural therapy, Motivation, Active, Passive

## Abstract

**Background:**

Lifestyle modification is widely recommended to kidney allograft recipients post transplantation due to the cardiometabolic risks associated with immunosuppression including new-onset diabetes, weight gain and cardiovascular events. However, we have no actual evidence that undertaking lifestyle modification protects from any adverse outcomes post transplantation. The aim of this study is to compare whether a more proactive versus passive interventional approach to modify lifestyle is associated with superior outcomes post kidney transplantation.

**Methods/design:**

We designed this prospective, single-centre, open-label, randomised controlled study to compare the efficacy of active versus passive lifestyle intervention for kidney allograft recipients early post transplantation. A total of 130 eligible patients, who are stable, nondiabetic and between 3 and 24 months post kidney transplantation, will be recruited. Randomisation is being undertaken by random block permutations into passive (*n* = 65, leaflet guidance only) versus active lifestyle modification (*n* = 65, supervised intervention) over a 6-month period. Supervised intervention is being facilitated by two dietitians during the 6-month intervention period to provide continuous lifestyle intervention guidance, support and encouragement. Both dietitians are accredited with behavioural intervention skills and will utilise motivational aids to support study recruits randomised to active intervention. The primary outcome is change in abnormal glucose metabolism parameters after 6 months of comparing active versus passive lifestyle intervention. Secondary outcomes include changes in a wide array of cardiometabolic parameters, kidney allograft function and patient-reported outcome measures. Long-term tracking of patients via data linkage to electronic patient records and national registries will facilitate long-term comparison of outcomes after active versus passive lifestyle intervention beyond the 6-month intervention period.

**Discussion:**

This is the first randomised controlled study to investigate the benefits of active versus passive lifestyle intervention in kidney allograft recipients for the prevention of abnormal cardiometabolic outcomes. In addition, this is the first example of utilising behaviour therapy intervention post kidney transplantation to achieve clinically beneficial outcomes, which has potential implications on many spheres of post-transplant care.

**Trial registration:**

This study was registered with the Clinical Trials Registry on 27 August 2014 (ClinicalTrials.org Identifier: NCT02233491).

## Background

Post-transplantation diabetes mellitus (PTDM) is a common medical complication after kidney transplantation and is associated with long-term morbidity, mortality and increased cost [1]. From a patient perspective, development of PTDM is cited by kidney transplant recipients as their biggest concern post transplantation after the risk of graft loss, death and cancer [2]. In the immediate postoperative setting over 85 % of kidney allograft recipients have evidence of significant hyperglycaemia [3] and it has been estimated that, using contemporary diagnostic criteria, up to 39 % of kidney allograft recipients will develop PTDM within the first year after transplantation [1]. PTDM is common because kidney transplant recipients have both transplant-specific (e.g. immunosuppression) and nonspecific (e.g. age, ethnicity, obesity) risk factors [4]. Therefore, strategies to attenuate this high risk for developing PTDM (and other cardiometabolic risk factors such as weight gain, hyperlipidaemia and hypertension) should be actively pursued after kidney transplantation to prevent adverse long-term outcomes, although evidence that providing this advice improves clinical outcomes is lacking.

In the general population, lifestyle modification has been shown to reduce the long-term risk of developing type-2 diabetes mellitus among high-risk patients (e.g. those with pre-diabetes). Tuomilehto et al. showed how the cumulative incidence of diabetes in a lifestyle intervention group was 11 % compared with 23 % in a control group of similar characteristics, resulting in a 58 % reduction in the risk of diabetes **(hazard ratio 0.4, 95 % confidence interval 0.3–0.7,*p*** **< 0.001)** [5**]**. In another study to support these findings, Pan et al. showed that diet, exercise and a diet-plus-exercise intervention was associated with a **relative risk of 0.69 (standard error 0.17,*p*** **< 0.03), 0.54 (standard error 0.01,*p*** **< 0.0005) and 0.58 (standard error 0.17,*p*** **< 0.005),** respectively, in the risk of developing diabetes compared with the control group [6]. Finally, Lindstrom et al. demonstrated a **reduced hazard ratio of 0.57 (95 % confidence interval 0.43–0.76,*p*** **= 0.00001)** secondary to lifestyle intervention [7]. Importantly, in this latter study the beneficial changes persisted after discontinuation of the intervention. Recent data has confirmed that lifestyle intervention is associated with **a reduced hazard ratio of 0.73 (95 % confidence interval 0.65–0.83,*p*** **< 0.0001)** in diabetes incidence during a mean follow-up of 15 years after participation in the 3-year Diabetes Prevention Programme [8], attenuating concern regarding long-term sustainability beyond interventional study periods.

While evidence to support lifestyle modification exists in the general population, it is important to appreciate the different pathophysiology associated with the development of type-2 diabetes mellitus versus PTDM. In a multicentre collaboration, Hecking et al. analysed glycaemic parameters comparing kidney allograft recipients with individuals from the general population (both normal subjects and those with type-2 diabetes mellitus), demonstrating disparate pathophysiology favouring more pancreatic beta-cell dysfunction in the transplant cohort with PTDM [9]. Despite these differences, clinical evidence from the general population is frequently extrapolated to kidney allograft recipients and current guidance recommending lifestyle modification for all kidney allograft recipients to attenuate their risk for PTDM is one example of this [10]. However, there is a growing consensus on the urgent need for transplant-specific clinical studies due to altered risk factor patterns and reverse epidemiology being commonly described in the renal failure population [11].

We have previously demonstrated the successful use of active lifestyle modification in the setting of kidney transplantation [12]. Kidney allograft recipients with abnormal postprandial glucose metabolism on an oral glucose tolerance test (OGTT) (impaired glucose tolerance and PTDM, *n* = 36) were selected to have active lifestyle modifications (dietitian referral, a graded exercise programme and weight-loss advice), while kidney allograft recipients with normal postprandial glucose metabolism (normal and impaired fasting glucose, *n* = 79) received leaflets advocating healthy lifestyle modifications alone. In this study, patients receiving active lifestyle intervention demonstrated a significant reduction in postprandial glucose levels (10.2 mmol/L to 8.7 mmol/L, *∆* = −15 %, *p* = 0.012) and improved reclassification of glycaemic status on reevaluation by OGTT 6 months later. By contrast, simple lifestyle modification advice by leaflets alone in the group with normal glucose tolerance resulted in a significant deterioration of postprandial glucose levels (5.9 mmol/L to 6.6 mmol/L, *∆* = +12 %, *p* = 0.001) and worse glycaemic status reclassification on follow-up OGTT. The limitations of this study were the lack of randomisation and noncomparable groups (with different metabolic profiles), meaning that a direct comparison of the benefits of active versus passive lifestyle intervention was not possible. Therefore, it is difficult to ascertain whether changes in glucose metabolism were directly linked to active versus passive lifestyle intervention.

To fill this gap in the literature, and to determine transplant-specific clinical evidence, this study has been designed as the first randomised controlled trial to assess the efficacy of active versus passive lifestyle intervention in a high-risk transplant cohort to attenuate the risk of abnormal glucose metabolism. In addition to being the first clinical trial of lifestyle intervention, this randomised controlled trial is also the first example of a behaviour change protocol being utilised as part of an interventional programme to alter behaviour post transplantation, which could have potential applications beyond lifestyle intervention alone for solid organ transplant recipients. Interventions to change health-related behaviours, such as diet and exercise, are usually complex, consisting of many interacting components [13]. Furthermore, they are often poorly reported in the research literature, limiting the possibility of identifying their effective ingredients. Therefore, attention has recently been paid to the standardised reporting of intervention content and the component behaviour change techniques (BCTs) [13, 14]. The development of a taxonomy of BCTs has allowed the use of meta-analysis and meta-regression to assess the effectiveness of behaviour change interventions designed to promote physical activity and healthy eating [15–17]. This work suggests that self-regulatory techniques congruent with Control Theory [18] are significantly more effective than interventions not including these techniques. These include encouraging the individual to decide to act (intention formation), prompting specific goal setting, providing feedback on performance and self-monitoring of behaviour, and reviewing previously set goals or intentions. These techniques were combined with two other effective strategies to support the behaviour change intervention. Firstly, ‘Node-link mapping’ (NLM) is a simple visual representation system for presenting behaviour change interventions, supported by a body of educational psychology and treatment research showing that its use is more effective than standard consultation techniques for improving the therapeutic alliance, increasing focus on key issues during the session, and improving outcomes [19]. It also has an evidence base in patients with poor reading skills or working in a language other than their first language [19]. Secondly, Social Behaviour and Network Therapy (SBNT) focusses on building social network support for behaviour change by assessing the patient’s level of support from family and friends and inviting key supportive others to assist with goal setting and monitoring. Evidence supporting the validity of this intervention has been published in the liver transplant population [20] and will be translated over to a kidney transplant cohort.

In summary, this randomised controlled trial will assess the efficacy of a novel behaviour change intervention technique to encourage active lifestyle modification in nondiabetic kidney allografts recipients who are at risk for PTDM and promises to fill an important gap in the literature to guide clinical management and patient counselling.

### Specific research questions

This study is designed to answer four important questions relevant to kidney transplant recipients who are at risk of developing PTDM:

1. Does lifestyle modification intervention improve glycaemic, cardiometabolic, clinical and physiological parameters in kidney allograft recipients?
2. Is active dietitian-led intervention (incorporating a behaviour therapy approach) more effective than passive leaflet-driven guidance?
3. Does active versus passive lifestyle modification improve patient-reported outcomes measures post kidney transplantation?
4. Are there long-term clinical advantages beyond the 6-month intervention period for either active or passive groups?

## Methods/design

### Study design

This is a prospective, single-centre, open-label, randomised controlled study comparing active versus passive lifestyle intervention post kidney transplantation. All eligible kidney allograft recipients who meet the inclusion/exclusion criteria will be invited to participate. The duration of the clinical intervention in the study will be 6 months, with electronic data-linkage follow-up extending out to 5 years post study participation.

### Study setting

Patients will be recruited from a single transplant centre located in Birmingham, England, and it is planned to recruit 130 nondiabetic kidney allograft recipients. Birmingham, and the greater West Midlands region which it serves, contains a diverse multicultural population and this study is keen to ensure that study participation is representative of the population we serve. Therefore, we will strive to ensure adequate representation from the Black, Asian and Minority Ethnic (BAME) communities. Our dietitians have excellent experience of working with the local demographics in Birmingham and tailored information for the various BAME cohorts will be available via both the active and passive lifestyle intervention arms.

### Inclusion and exclusion criteria

#### Inclusion criteria

- Aged 18 and over
- Kidney allograft only
- Functioning allograft (not on dialysis)
- Between 3 and 24 months post kidney transplantation

#### Exclusion criteria

- Multiorgan transplant recipient
- Preexisting known diabetes
- Pregnancy
- Physically unable to undertake exercise assessments

### Study protocol and interventions

Potential participants will be identified through computerised search of medical records on patients attending clinic and from clinicians in charge of outpatient services. Relevant clinic lists will be reviewed on a weekly basis and potential participants identified (first approach will be from medical team or renal research nurse). Potential participants will be given the opportunity to reflect on the information given both verbally and within the written information sheet. No fixed time is specified for the length or timing of this prior interview as it will be dependent upon the patient’s understanding of their underlying disease, transplantation and complications, as well as their understanding of the research project.

**Fig. 1:**
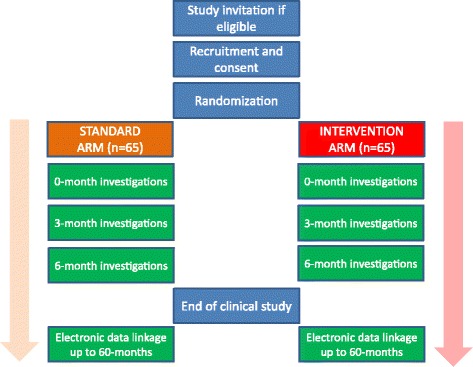
Trial design for the CAVIAR study showing randomisation arms comparing active versus passive lifestyle intervention in 130 nondiabetic kidney allograft recipients

**Fig. 2:**
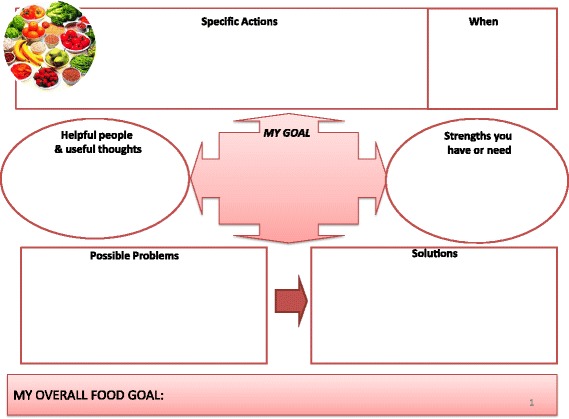
Intervention slide outlining patient dietary goals used to support the behaviour change element of the active lifestyle intervention

**Fig. 3:**
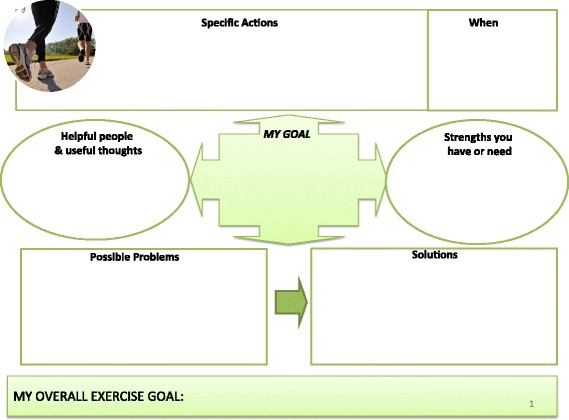
Intervention slide outlining patient exercise goals used to support the behaviour change element of the active lifestyle intervention

**Fig. 4:**
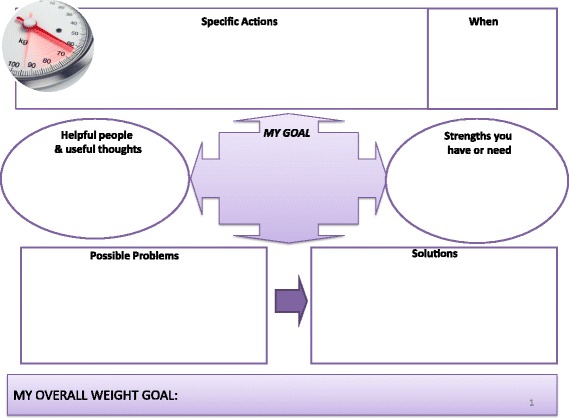
Intervention slide outlining patient weight goals used to support the behaviour change element of the active lifestyle intervention
- *Passive control group*. This group will be counselled about the risks of glucose intolerance and will receive leaflets outlining lifestyle modification advice. The leaflets include advice on healthy eating, exercise and the importance of weight loss. However, there will be no dietitian referral or focussed exercise and weight-loss monitoring review and no behaviour therapy intervention. Follow-up will be at routine clinic visits only where lifestyle modification advice will be reinforced as per usual clinical practice.

Kidney allograft recipients who fulfil the eligibility criteria and give informed consent will be subsequently randomised into active versus passive intervention arms (at which point an OGTT is performed in each patient *after* randomisation and *before* intervention) (see Fig. 1). Randomisation will be facilitated through www.sealedenvelope.com, using random permuted blocks within strata to balance numbers and characteristics, into one of the following lifestyle intervention groups (*n* = 65 for each intervention group, total 130 participants):

- *Active intervention group*. This group will receive active lifestyle modification intervention and will consist of dietitian referral, a graded exercise programme and weight-loss advice. Each patient will have four face-to-face appointments with the dietitian (lasting 45–60 min) at baseline, day 30, day 60 and day 120. Brief telephone reviews will be conducted between appointments (2–4 weeks after each face-to-face appointment) to review progress and provide additional support during the 6-month active intervention period (some appointments could be substituted with telephone support if wished by patient). Patients will have their diet reviewed by a dietitian and healthy eating advice will be given based upon guidelines issued by Diabetes UK [21]. The guidelines recommend a diet containing less fat and more fibre, based upon a meal framework of 50 % carbohydrate, 25 % protein (meat, fish, beans) and 25 % fibre (fruit and vegetables). Patients will be advised to keep food diaries to monitor compliance with initiated changes and will be followed up by the dietitians prospectively (using face-to-face appointments and telephone reviews) to monitor progress and reinforce the advice (in addition to routine clinic visits).
- In addition, a graded exercise programme will be encouraged to increase physical activity (e.g. endurance exercise such as walking, jogging, swimming) and an exercise diary will be encouraged to monitor compliance. To establish and prospectively monitor exercise habits we will perform Incremental Shuttle Walk Tests. Validated surveys to assess physical capability and activity (Duke Activity Status Index and GP Physical Activity Questionnaire, respectively) will be performed at each nurse-led assessment.
- The dietitian will be supported by our collaboration with a senior lecturer and consultant in addiction psychiatry, who has a recognised expertise in behaviour change therapy, and will support the behaviour therapy component of the active intervention arm. The behaviour change intervention will be developed in conjunction with the dietitians, and will utilise NLM tools developed from a Public Health England manual [22]. Based on the research evidence summarised above, it will include the following BCTs:
  1. Provide information on the consequences of suboptimal diet and exercise levels on health in general
  2. Provide specific feedback of personalised information (body mass index, body fat percentage, waist/hip ratio) and comparison with the healthy range
  3. Prompt intention formation, i.e. encouraging the patient to make a resolution to change their diet or level of exercise
  4. Set specific goals around diet, exercise and weight (see Figs. 2, 3 and 4)
  5. Set graded tasks around the achievement of patient goals
  6. Encourage self-monitoring of goals through food and exercise diaries and other Node-link maps
  7. Regular review of specific behavioural goals, and reinforcement of progress through praise and encouragement
  8. Review of social support available from personal network of family and friends, and linking support to the achievement of specific goals.

After 6 months of either intervention, both groups will undergo a repeat OGTT to assess any change in glycaemic status and metabolic physiological parameters. If any kidney allograft recipient develops PTDM during the course of the study, then they will be treated as per protocol (lifestyle modification advice with glucose-lowering therapy based upon individualised clinical assessment).

### Study outcomes

#### Primary endpoint

1. Difference in insulin secretion, sensitivity and disposition index

#### Secondary endpoints

1. Incidence of PTDM
2. Incidence of impaired glucose tolerance
3. Incidence of impaired fasting glucose
4. Commencement of glucose-lowering therapy
5. Difference in glycated haemoglobin (HbA1c)
6. Change in weight, body mass index, waist/hip ratio, triceps skin-fold thickness
7. Change in bioimpedance parameters (fluid, fat and muscle tissue mass and indexes)
8. Change in blood pressure and lipid profile
9. Change in physical activity (Incremental Shuttle Walk Tests, Duke Activity Status Index and GP Physical Activity Questionnaire, respectively)
10. Change in psychological wellbeing (using two of three instrumental tools which have been validated in kidney transplant populations):
  EQ5D – quality of life and health status [23]
  Beck Depression Inventory (BDI-II) – specific tool for depression* [24]
  Situational Motivation Scale (SMS) – specific tool for assessment of situational motivation [25]
11. Long-term clinical outcomes (patient survival, allograft survival, cardiac events, weight, etc) via electronic tagging to the electronic patient records at University Hospitals Birmingham (via Patient Registration number) and national registries (via NHS number)

*Patients will be offered leaflets with the clinical psychology department’s contact details if self-harm is reported.

#### Safety endpoints

1. Change in creatinine
2. Change in proteinuria (urine albumin-creatinine ratio)

### Flowchart of investigations

#### Clinical data

Demographic data will be collected for each individual participant at the beginning of the study to record baseline clinical details. Pertinent clinical data (e.g. weight, waist/hip ratio, triceps skin-fold thickness, blood pressure, bioimpedance parameters (fluid, fat and muscle mass), etc.) will be collected at baseline and at follow-up at selected study points as demonstrated in Table 1.

**Table 1:** Flowchart of study investigations

Investigations	Days post recruitment					Years post recruitment		
	0	60	90	120	180	1	3	5
	Randomisation		Mid-way point		Post intervention	Post intervention	Post intervention	Post intervention
Dietitian review with clinical psychology input (if active arm)	X	X		X				
Standard blood and urine tests^a^	X	X	X	X	X			
Baseline demographics^b^	X							
Clinical assessment^c^	X		X		X			
Patient-reported outcomes^d^	X		X		X			
Glucose	X	X	X	X	X			
Insulin	X		X		X			
Diabetes/obesity immunoassay^e^	X		X		X			
Triglycerides	X		X		X			
Oral glucose tolerance test	X				X			
HbA1c	X		X		X			
Electronic capture of clinical outcomes^f^						X	X	X

^a^Routine clinical blood and urine tests include renal function, tacrolimus levels, liver function tests, full blood count, urine albumin-creatinine ratio, etc.

^b^Extensive baseline data including cause of end-stage kidney disease, dialysis modality, dialysis duration, family history of diabetes, gestational diabetes, hepatitis C status, etc.

^c^Blood pressure, waist/hip ratio, weight, height, body mass index, bioimpedance (fluid, fat and muscle mass/index estimation), etc.

^d^Patient-reported outcomes (physical activity and psychological wellbeing as highlighted above)

^e^C-peptide, ghrelin, gastric inhibitory peptide (GIP), glucagon-like peptide-1 (GLP-1), glucagon, leptin, total plasminogen activator inhibitor-1 (PAI-1), resistin, visfatin and adiponectin

^f^Long-term clinical outcomes (patient survival, allograft survival, cardiac events, weight, etc) via electronic tagging to University Hospital Birmingham’s electronic patient records (via Patient Registration number) and national registries (via NHS number)

#### Biochemical data

Biochemical tests, to be done at baseline and follow-up (see Table 1) will include: plasma total cholesterol, low-density lipoprotein (LDL) and high-density lipoprotein (HDL) cholesterol, triglycerides, fasting glucose, urea and electrolytes, creatinine, full blood count, C-reactive protein (CRP), HbA1c, insulin and a range of diabetes/obesity biomarkers via a multiplex immunoassay (includes C-peptide, ghrelin, GIP, GLP-1, glucagon, leptin, total PAI-1, resistin, visfatin and adiponectin). The OGTT will obtain fasting blood samples for glucose in addition to routine clinic bloods after an overnight 12-h fast. Patients will then be administered 75 g of glucose (113 mL of Polycal®) with postprandial samples taken 2 h after glucose administration. The results of the test will be classified by current International Consensus recommendations for the diagnosis of PTDM [10], which mirror guidance in the general population for the diagnosis of diabetes [26].

#### Metabolic parameter assessments

Venous blood sampling from both baseline and follow-up OGTTs will be used to determine changes in insulin sensitivity, insulin secretion and the disposition index (interrelationship between both insulin sensitivity and secretion). The following formulae will be utilised for determination of these parameters of glucose metabolism:

$$ \underline {Insulin\kern0.5em  secretion}=HOM{A}_{sec}= Insuli{n}_0\times \kern0.5em \left[ 3.33\kern0.1em /\kern0.1em \left( glucos{e}_0- 3.5\right)\right] $$

$$ \begin{array}{ll}&{Insulin\kern0.5em  sensitivity}= McAuley's\kern0.5em  index= exp\\ &\qquad\qquad\qquad\qquad\qquad \times\left[\mathit{ 2. 6 3}-\left( \mathit{0. 2 8}\kern0.5em \times \kern0.5em  In\kern0.5em \left\{ {\mathit{insulin}}_{\mathit{0}}/ \mathit{6. 945}\right\}\right)\right.\\&\qquad\qquad\qquad\qquad\qquad-\left.\left(\mathit{ 0. 31}\kern0.5em \times \kern0.5em  In\kern0.5em  {\mathit{trigycerides}}_{\mathit{0}}\right)\right]\end{array} $$

$$ \begin{array}{l}\underline {Disposition\kern0.5em  index}=HOM{A}_{sec}\times McAuley\mathit{\hbox{'}}s\kern0.5em  index= In suli{n}_0\times \left[ 3.33\kern0.2em /\kern0.1em \left( glucos{e}_0- 3.5\right)\right]\kern0.5em \times \kern0.5em \\ {} exp\kern0.5em \left[ 2.63-\left( 0.28\kern0.5em \times \kern0.5em  In\kern0.5em \left\{ insuli{n}_0/ 6.945\right\}\right)-\left( 0.31\kern0.5em \times \kern0.5em  In\kern0.5em  trigyceride{s}_0\right)\right]\end{array} $$

Surrogates for insulin sensitivity [27] and disposition index [28] have previously been validated and reported in the context of kidney transplantation. In addition to allowing us to determine the difference between active versus passive lifestyle intervention, comparison of baseline and follow-up glucose metabolic parameters will provide some insight into underlying pathophysiological mechanisms that lead to progression of abnormal glucose metabolism post transplantation.

#### Electronic data capture

Patients will be consented to electronic data capture of clinical parameters (e.g. weight, body mass index, creatinine, etc) from the electronic patient records of University Hospitals Birmingham at 1, 3 and 5 years post recruitment (utilising individual unique Patient Registration number). In addition, clinical outcomes (e.g. patient survival, allograft survival, hospitalisation, cardiac events, etc) will be electronically captured from Hospital Episodes Statistics for the same time points (utilising NHS number). This will allow long-term data capture without direct patient involvement and allow us to investigate the long-term effects of study interventions beyond the initial study intervention period.

#### Sample size

The principle parameters being examined in this study are changes in insulin sensitivity and insulin secretion. Our power calculation was performed with the assumption of a 20 % dropout rate and on the basis of an intention-to-treat analysis. We anticipate changes in the primary outcome measure from 5 % in the control group and 25 % in the intervention group. These figures are based on intrasubject variability of 25 % for insulin secretion and 20 % for insulin sensitivity, as previously utilised in a different randomised controlled trial in a post-transplantation setting [29].

Therefore, if we assume 80 % of the control group will demonstrate a 5 % change in the primary outcome measure (and 20 % dropouts demonstrate no change), then the average change in the control group is 4 %. Similarly, if we assume 80 % of the intervention group will demonstrate a 25 % change in the primary outcome measure (and 20 % dropouts demonstrate no change), then the average change in the intervention group is 20 %.

To detect this difference of 16 % change (assuming standard deviation of change is 25 %), we calculate that a total of 130 patients are required (65 per randomised arm) for 95 % power (assuming a 5 % significance level and a two-sided test) for a high-powered sample size.

#### Statistical analysis

Normality of data will be assessed using the Kolmogorov-Smirnov tests. The paired sample *t* test and the Wilcoxon signed rank test, for parametric and nonparametric data, respectively, will be used to compare the means of two variables from a single group. Comparison of data between groups will be made using unpaired Student’s *t* tests and the Mann-Whitney test for parametric and nonparametric data, respectively. Categorical data will be analysed using Pearson’s or Spearman’s test as appropriate.

We will use Cox proportional hazards regression to analyse survival outcomes. Additionally, for all primary and secondary analyses, we will carry out multivariable analyses using generalised linear models (adjusting for stratification factors). In this case, we will use the corresponding odds ratio to evaluate the adjusted size of the differences between proportions, by transforming back the estimated odds ratio associated with the treatment group into absolute differences in proportions. If the results of the analysis of the primary outcome are significantly affected, the adjusted effects will be taken as final. A *p* value <0.05 will be considered significant in the statistical analysis.

#### Withdrawal of subjects

Individual recruits can withdraw at any time for any reason. Should a patient decide to withdraw from the study, all efforts will be made to report the reason for withdrawal as thoroughly as possible. Participants who wish to withdraw from treatment will be asked to confirm whether they are still willing to provide study-specific data and samples for scientific laboratory analysis according to the trial protocol. In addition, should a patient withdraw, efforts will be made to obtain follow-up data with the permission of the patient (e.g. electronic data capture).

## Discussion

Despite the importance of PTDM as a cause of morbidity, mortality, increased cost and patient anxiety, there is a paucity of randomised controlled trials exploring interventions that can attenuate the development of diabetes in a metabolically high-risk cohort like kidney allograft recipients. While strategies to attenuate the high risk for developing PTDM (and other cardiometabolic risk factors) should be actively pursued after solid organ transplantation, we have no evidence that these interventions are beneficial (either in the short or long term) and this evidence is extrapolated from the general population. However, transplant-specific data is important as the cardiometabolic milieu of kidney transplantation is different from the general population due to immunosuppressant side effects, weight gain and fatigue being more common post transplantation. We should, therefore, be hesitant to simply translate guidance from general to kidney transplant recipient cohorts. In addition, the metabolically high-risk environment after kidney transplantation may justify a more active approach to deliver lifestyle modification intervention. While lifestyle modifications are recommended as part of existing PTDM International Consensus recommendations [10], they lack a clinical evidence base post transplantation and potential efficacy has been translated from outcome data in the general population. In addition, no recommendation is made with regards to whether intervention should be active or passive. Previous work has shown that active lifestyle intervention (dietitian-led) improved glucose metabolism in kidney allograft recipients with impaired glucose metabolism, while by contrast passive lifestyle intervention (leaflet advice) led to deterioration in glucose metabolism in kidney allograft recipients with normal glucose metabolism [12]. The limitations of this study were that it was nonrandomised and comparison of active versus passive lifestyle intervention was not performed in a similar cohort of patients. This randomised controlled study will be the first clinical trial to explore the benefits of active versus passive lifestyle intervention post kidney transplantation.

One of the great strengths of this study design is the specialist involvement to support behaviour therapy change in study recruits to encourage active lifestyle intervention. This is an important aspect of care as the key role of cognitive processes in the success or failure of lifestyle modification is well-documented [30]. From a transplantation perspective, there is a paucity of data with regards to the psychological aspects of receiving a kidney allograft. Evidence suggests that kidney allograft recipients experience a range of positive and negative emotions after kidney transplantation including guilt, fear and gratefulness [31]. Psychological conflicts have been shown to affect both motivation and behaviour to undertake lifestyle modification post solid organ transplant recipients [32]. No randomised controlled trial in kidney transplantation has ever been published evaluating the efficacy of psychosocial intervention through behaviour therapy and the potential application of this study to other domains of post-transplant care (e.g. medication adherence, anxiety, depression, etc) are significant.

In summary, this randomised controlled trial assessing the efficacy of a multidisciplinary approach to encourage lifestyle interventions on glucose metabolism has been designed to answer unresolved questions in an important cohort of patients at high risk for developing PTDM. If the aims of this project are realised, then it will provide a clinical evidence base to support active lifestyle interventions as a standard part of the post-transplant care delivered to all kidney allograft recipients and address one of the many outstanding research questions in kidney transplantation. It will also support the reorganisation of service delivery with multidisciplinary care support by allied health professionals to ensure that patient education through active lifestyle intervention is actively pursued for kidney allograft recipients.

### Trial status

Patient recruitment started in August 2015 and at the time of submission, 48 kidney allograft recipients have been recruitment and randomised to active or passive lifestyle intervention. The clinical trial is on target to complete recruitment by 2017.

### Endnotes

In the transplantation literature, post-transplantation diabetes mellitus (PTDM) has been recommended to replace the previous terminology of new-onset diabetes after transplantation (NODAT). While we have utilised the up-to-date terminology to reflect this shift in emphasis, readers may find some continued publications citing the older term NODAT in the literature.

## Abbreviations

BAME: Black, Asian and Minority Ethnic
BCT: Behaviour change technique
BDI: Beck Depression Inventory
EQ5D: EuroQol 5D
GIP: Gastric inhibitory peptide
GLP-1: Glucagon-like peptide-1
HbA1c: Glycated haemoglobin
HOMA: Homeostasis model assessment
NLM: Node-link mapping
OGTT: Oral glucose tolerance test
PAI-1: Plasminogen activator inhibitor-1
PTDM: Post-transplantation diabetes mellitus
SBNT: Social Behavioural and Network Therapy
SMS: Situational Motivational Scale
